# Aging Characteristics of Bitumen from Different Bituminous Pavement Structures in Service

**DOI:** 10.3390/ma12030530

**Published:** 2019-02-10

**Authors:** Xiaofeng Wang, Haoyan Guo, Bo Yang, Xingwen Chang, Chenguang Wan, Zhenjun Wang

**Affiliations:** 1 Research and Development Center of Transport Industry of Technologies, Materials and Equipments of Highway Construction and Maintenance, Zhengzhou 450052, China; wangxf0351@sina.com (X.W.); yangbohnrbi@foxmail.com (B.Y.); changxw1963@aliyun.com (X.C.); wancg1989@aliyun.com (C.W.); 2 Henan Engineering Research Center of Intelligent Highway Big Data, Zhengzhou 450052, China; 3 School of Materials Science and Engineering, Chang’an University, Xi’an 710061, China; hyguo@chd.edu.cn; 4 Engineering Research Center of Pavement Materials, Ministry of Education of P.R. China, Chang’an University, Xi’an 710064, China

**Keywords:** aging characteristics, bitumen, bituminous pavement structures, in service, microscopic characterizations

## Abstract

The aging of bitumen seriously affects the service life of bituminous pavements. At present, there are many related researches on bitumen aging, but most of them focus on aging endured in indoor surroundings and conditions. Therefore, the conclusions obtained cannot reflect the actual aging changes of bitumen in bituminous pavements in service. In order to study the comprehensive aging process and mechanism of bitumen under the influence of service, we studied bridge deck, traffic lane, and ramp with bituminous pavement structures in service. The bitumen samples obtained from the core samples in different bituminous pavement structures were characterized by gel permeation chromatography (GPC), Fourier transform infrared spectroscopy (FTIR), dynamic shear rheometer (DSR), and fluorescence microscope (FM). The aging degree of different bitumen was analyzed, and conclusions were drawn on changes to bitumen aging from different pavement structures. The results showed that the aging degree of bitumen from the upper layer was the most serious, the aging degree of bitumen at the middle layer was weaker than that of bitumen from the upper layer, and the aging degree of bitumen from the bottom layer was the weakest for the different bituminous pavement structures. The aging of bitumen mainly occurred due to oxygen absorption. After aging, viscoelastic components of bitumen changed, and bitumen became harder. The macromolecule of bitumen could be divided into small molecules, and the small molecular weight of bitumen became large. The styrene-butadiene-styrene (SBS) modifier in the modified bitumen became granular after aging, and it appeared as a single phase in bitumen. The aging changes characterized by different analytical methods showed that the aging degree of bitumen from different layers of bituminous pavement structures in service was different. Effective measures should therefore be taken in time to decrease further aging of bitumen from the upper layer of bituminous pavements due to its inevitable early aging in service.

## 1. Introduction

Bituminous pavements face comprehensive aging effect from vehicle load and the natural environment (such as temperature, oxidation, light, rainwater), which can result in the aging of bitumen in the bituminous mixture. Bitumen can become harder and brittle after a series of physical and chemical changes during the aging process [1]. In addition, the aging of bitumen can directly affect the service life of pavements. Macroscopic changes during bitumen aging include a decrease in penetration and ductility and an increase in the softening point. This is accompanied by a reduction in other properties of bituminous pavements, such as anti-rutting, water damage resistance, and crack resistance [2,3,4]. Therefore, the aging of bitumen has become one of the main factors affecting the service life of bituminous pavement structures.

In recent years, many researchers have studied the aging process and mechanism of bitumen [5,6,7]. Wang et al. [8] studied the aging process of polymer–bitumen composite systems by chemical functional group, molecular size, and rheological characteristics. Lana et al. [9] evaluated the effects of aging on micro mechanical and chemical properties of foam warm mix bitumen using gel permeation chromatography (GPC) and atomic force microscope (AFM). Bowers et al. [10] studied the fusion degree of aged bitumen and new bitumen in reclaimed bituminous pavements using GPC and Fourier transform infrared spectroscopy (FTIR). Menapace et al. [11] processed bitumen using an accelerated aging tester and analyzed the chemical composition of aged bitumen by FTIR and X-ray photoelectron spectroscopy. Lee et al. [12] tested the variations in large molecular particle size (LMS) of rubber-modified bitumen before and after aging using GPC. Wang et al. [13] studied the surface morphology of five kinds of bitumen in different aging situations using AFM. Chen et al. [14] used AFM to study the aging mechanism of bitumen and found that the light component content of bitumen was greatly reduced during the aging process. Dai et al. [15] processed styrene-butadiene-styrene (SBS)-modified bitumen using rotating film oven aging and pressurized aging vessel (PAV) and then obtained nanoscale morphology and rheological properties of SBS-modified bitumen after aging using AFM and the rheometer test. Zhang et al. [16] studied the impact of various aging methods on the physical properties and chemical composition of SBS-modified bitumen using the film furnace test, PAV, and ultraviolet (UV) radiation.

In addition, there have been some innovations in the study of bitumen aging, which are reflected in both devices and testing methods. Some scholars have developed new devices to study the aging mechanism of bitumen under different aging conditions, while others have applied traditional testing methods or instruments in their studies. Ye et al. [17], for example, developed a strong UV aging box with reference to the actual UV light intensity of field bituminous pavements. Yu et al. [18] used AFM technology to quantitatively analyze the morphology, adhesion, and modulus of traditional rotating film oven aging + PAV-aged bitumen and all weather-aging bitumen. Yan et al. [19] found that the rolling film oven test was not suitable for polymer-modified bitumen because the high viscosity polymer-modified bitumen did not roll in the glass bottle during the test. Hou et al. [20] used the spectrophotometric method to determine the aging characteristics of bitumen.

Researchers are also increasingly using simulation techniques to analyze the aging mechanism of bitumen [21,22,23]. Zhang et al. [24], for example, used the finite element model to study the aging process of bitumen mortar and studied the effect of aging on fatigue resistance. Chen [25] analyzed changes in the quality of bitumen components before and after aging using thermogravimetric analysis and established a dynamic model between quality and aging time. Xu et al. [26] used molecular dynamics to simulate the oxidative aging reaction of bitumen. Ding et al. [27] established a corresponding molecular model of components of original and aged bitumen by studying the functional group distribution of unaged and aged bitumen using liquid chromatography transformation with GPC and FTIR.

Some scholars have considered the aging conditions of indoor tests to be unsuitable for simulating the true aging mechanism of roads in service and have therefore proposed an aging test of bitumen under the coupling of various conditions. Ying et al. [28] put up a concept of bitumen coupling aging and discussed the reduction in bitumen performance. Liu et al. [29] analyzed the changes in viscosity and microscopic composition of bitumen after indoor, accelerated aging progress and after natural aging using dynamic shear rheometer (DSR) and GPC and concluded that there is still some difficulty in simulating the true aging mechanism of bitumen in the laboratory.

In summary, the study of bitumen aging at present is mostly limited to indoor, simulated aging, which is obviously different from the actual aging of bituminous pavements under complex service conditions. The differences in the aging of different structural layers of bituminous pavements and aging characteristics are rarely distinguished. Therefore, in order to study the actual aging characteristics and aging degree of bitumen from different structural layers in bituminous pavements, three kinds of bituminous pavement structures—bridge deck, traffic lane, and ramp—under different service condition were selected in this study. The bitumen samples obtained from the core samples in different bituminous pavement structures were characterized by GPC, FTIR, DSR, and fluorescence microscope (FM). The aging degree of different bitumen was analyzed, and conclusions were drawn on changes to bitumen aging from different pavement structures. The conclusions obtained in this work about the physical and chemical changes in the aging process of bituminous pavements with different structures are expected to provide theoretical and technical support for the improvement of the antiaging ability and durability of bituminous pavement engineering in service.

## 2. Materials and Methods

### 2.1. Materials

#### 2.1.1. Bituminous Pavement Structures in Service

Bridge deck, traffic lane, and ramp with bituminous pavement structures were used in this work. The thickness of the bridge deck pavement structure was 5 cm AC-13 I fine-grained bitumen mixture + 5 cm AC-16 I medium-grain bitumen mixture, and the total thickness of the bituminous pavement structural layer in the bridge deck was 10 cm. The thickness of the bituminous pavement structural layer in the traffic lane was 4 cm AK-16A medium-grain asphalt mixture + 7 cm AC-20 I medium-grain asphalt mixture + 7 cm AC-25 I coarse-grain asphalt mixture, and the total thickness of the bituminous pavement layer at the traffic lane was 18 cm. The thickness of the bituminous pavement structural layer in the ramp was 3 cm AK-16A medium-grain asphalt mixture + 5 cm AC-20 I medium-grain asphalt mixture + 5 cm AC-25 I coarse-grain asphalt mixture, and the total thickness of the bituminous pavement layer in the ramp was 13 cm.

#### 2.1.2. Core Sample Selection of Bituminous Mixture

In accordance with the Field Test Methods of Subgrade and Pavement for Highway Engineering (JTG E60-2008), the core samples of the bituminous mixture from different pavement structures were obtained with a core drilling rig machine, and the samples were cylindrical specimens with a diameter of 150 mm. The descriptions of different bituminous pavement structures and the core samples are shown in Table 1.

### 2.2. Methods

#### 2.2.1. Bitumen Extraction

In this work, the centrifugal separation method (T 0722-1993) in the Standard Test Methods of Bitumen and Bituminous Mixtures for Highway Engineering in China (JTG E20-2011) was used to extract the bitumen solution from the core sample. The steps were as follows: (1) place the core sample in an oven and heat it to a loose state; (2) pour the sample and trichloroethylene into the centrifugal separator, soak for 30 min, and wait for bitumen to dissolve completely; (3) start the centrifuge, gradually increasing the rotational speed to 3000 r/min, and flow bitumen solution into the recycled bottle through the discharge port; (4) add clean trichloroethylene to the centrifuge and repeat step (3) until bitumen solution turns pale yellow. 

Next, the Abson method (T 0726-2011) in the Standard Test Methods of Bitumen and Bituminous Mixtures for Highway Engineering in China (JTG E20-2011) was used to recover the bitumen. The steps were as follows: (1) inject the extract in the recycled bottle into the centrifuge tube and remove the mineral powder in the extract by high-speed centrifugation; (2) inject the extract into the distillation flask and place the distillation flask in the distillation unit; (3) distill the trichloroethylene out of the extract under the conditions of oil bath and CO_2_ atmosphere and control the temperature in the distillation flask to 160–166 °C; (4) supply CO_2_ gas for 5 minutes continuously after the trichloroethylene solvent stops dropping to avoid secondary aging of bitumen; (5) obtain and analyze the recycled bitumen.

#### 2.2.2. GPC Test

GPC is an accurate and efficient method to classify polymers by molecular weight. This method is convenient and intuitive and is widely used in the study of molecular weight distribution of polymers. The test steps used in this work were as follows: (1) wash 10 mL bottles with tetrahydrofuran (THF); (2) weigh 20 mg of extracted bitumen sample into the 10 mL bottle and mark them; (3) drop 10 mL THF to the bottle using a dropper, cover the lid, shake well and stand, and wait for the bitumen sample to dissolve in THF completely; (4) filter with 0.45 um sieve to eliminate influences of other particles on bitumen; (5) absorb 0.4–0.5 mL filtrate with tube, inject the filtrate into the GPC instrument, and carry out the test; (6) analyze the obtained results with software. In this test, the temperature was set at 35 °C, the flow rate was 1.0 mL/min, and the concentration of the sample solution was 2.0 mg/mL.

**Figure 1:**
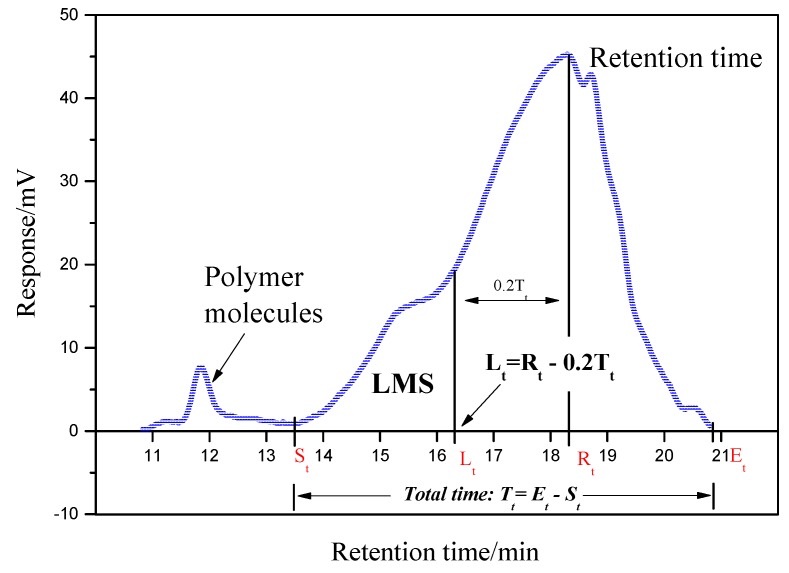
Gel permeation chromatography (GPC) analysis diagram of bitumen.

A GPC diagram of bitumen is shown in Figure 1 [30]. According to Reference [30], the peak time is labeled as the retention time (*R_t_*), LMS time (*L_t_*) can be determined by subtracting a fixed time interval from *R_t_*, and the fixed time interval is given as 0.2 of total time (*T_t_*). The total time can be calculated by subtracting the starting time (*S_t_*) from the ending time (*E_t_*). The location of *L_t_* is *S_t_* + 12/30*T_t_*. The area value of LMS below the curve can be calculated by a special software after the *L_t_* value is determined.

The molecular weight of bitumen can change during the aging process. Therefore, the aging degree of bitumen can be analyzed by the change in the molecular weight of bitumen. The average molecular weight of polymer was calculated using Equations (1)–(4) [31].

(1) $$\bar{M_{w}}\left(polymer\right)=\sum w_{i}M_{i}$$

(2) $$\bar{M_{n}}\left(polymer\right)=\sum n_{i}M_{i}$$

where,

(3) $$w_{i}=\frac{W_{i}}{\sum_{i}W_{i}}$$

(4) $$n_{i}=\frac{N_{i}}{\sum_{i}N_{i}}$$

where $\bar{M_{w}}\left(polymer\right)$ is the weight-average molecular weight, g/mol; $\bar{M_{n}}\left(polymer\right)$ is the number-average molecular weight, g/mol; *w_i_* and *n_i_* are the weight fraction and the number fraction, respectively, which can be calculated by Equations (3) and (4); *M_i_* represents the molecular weight of each molecular fraction, g/mol; *W_i_* is the weight of the fraction with molecular weight of *M_i_*, g; and *N_i_* represents the number (molar number) of each molecular fraction, mol.

#### 2.2.3. FTIR Test

FTIR can be used to analyze the variety and content of various chemical bonds in bitumen. The test steps in this work were as follows: (1) clean the sample bench with THF and alcohol; (2) place about 1 g bitumen sample on the sample bench, facing the diamond bit; (3) press the knob to make the diamond bit compact the bitumen sample and start the test; (4) obtain the test result. The temperature for the test was 20 °C. The spectra were collected in the wave number range from 4000 cm^−1^ to 500 cm^−1^ at the resolution of 4 cm^−1^.

The content of certain functional groups in SBS-modified bitumen usually changes to some extent during the aging process, especially for some oxygen-containing groups, such as carbonyl and sulfoxide functions, which appear as a change in characteristic peaks in the infrared spectrum. Of course, there are also groups that are not affected by aging in the aging process, and their content and peaks of characteristic peaks in the infrared spectrum do not change, such as C–H. Therefore, according to References [8,32], the aging degree of bitumen can be quantitatively analyzed by Equations (5)–(7).

(5) $$L_{\left(-CH=CH-\right)}=\frac{Area\left(966{\mathrm{cm}}^{-1}\right)}{Area\left(1376{\mathrm{cm}}^{-1}\right)}$$

(6) $$L_{\left(S=O\right)}=\frac{Area\left(1030{\mathrm{cm}}^{-1}\right)}{Area\left(1376{\mathrm{cm}}^{-1}\right)}$$

(7) $$L_{\left(C=O\right)}=\frac{Area\left(1700{\mathrm{cm}}^{-1}\right)}{Area\left(1376{\mathrm{cm}}^{-1}\right)}$$

#### 2.2.4. DSR Test

DSR (GEMINI 2, Malvern Instruments Co., Ltd., Malvin, UK) was used to analyze the viscoelastic behavior of bitumen. The test steps in this work were as follows: (1) heat bitumen and then pour it into mold; (2) demold after cooling and wait for testing; (3) place the sample on a plate with diameter of 25 mm and wait for the sample temperature to rise to 64 °C for at least 10 min; (4) lower rotation axis of DSR to adjust gap to 1 mm and carefully trim out bitumen beyond the edge of the plate; (5) start the test and obtain the testing results with complex shear modulus (G*) and phase angle of bitumen samples. The method used in this test was frequency sweep with frequency ranges of 0.1–10.0 Hz. Each sample was tested three times, and the average value was adopted as the testing result.

#### 2.2.5. FM Test

FM is used to analyze microstructures of bitumen in different aging stages. FM (LW300LFT, Xi’an Cewei Photoelectric Technology Co., Xi’an, China) is an instrument that uses ultraviolet light as a light source to irradiate the detected object and make it fluorescent. Then, the shape and location of the sample can be observed under the microscope. The experimental steps used in this work were as follows: (1) heat bitumen samples; (2) coat thin bitumen samples on microscope slide; (3) observe the coated slide under FM and obtain pictures of the tested samples. The test was carried out at a temperature of 20 °C.

## 3. Results and Discussion

### 3.1. Aging Degree Analyses of Bitumen

#### 3.1.1. Changes in Molecular Weight

**Figure 2:**
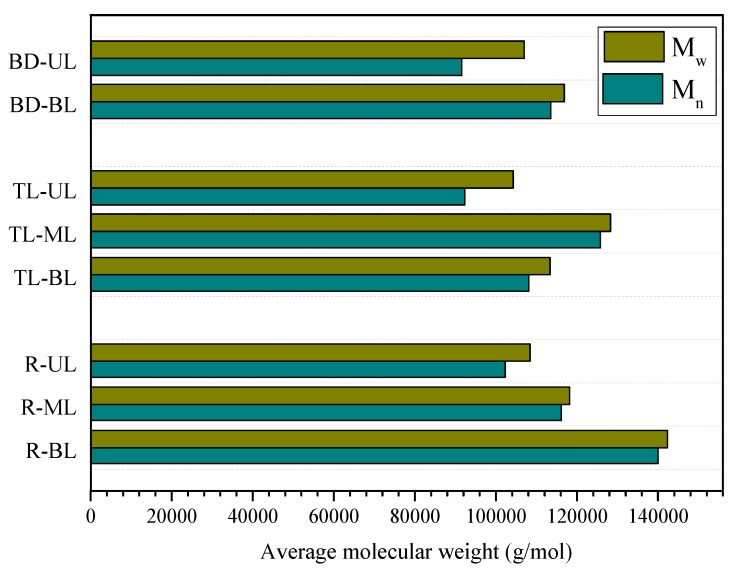
The *M_w_* and *M_n_* of bitumen after aging.

The number-average molecular weight (*M_n_*) and weight-average molecular weight (*M_w_*) of the polymer from the aged bitumen samples are shown in Figure 2. *M_w_* and *M_n_* can reflect the molecular weight of the tested samples. Polydispersity index (PDI) represents the molecular weight distribution width, which can be calculated by Equation (8). The PDI of the following were calculated: bitumen at bridge deck, upper layer of bituminous pavement (BD-UL); bridge deck, bottom layer of bituminous pavement (BD-BL); traffic lane, upper layer of bituminous pavement (TL-UL); traffic lane, middle layer of bituminous pavement (TL-ML); traffic lane, bottom layer of bituminous pavement (TL-BL); ramp, upper layer of bituminous pavement (R-UL), ramp, middle layer of bituminous pavement (R-ML); and ramp, bottom layer of bituminous pavement (R-BL). The results were 1.1654, 1.0264, 1.1263, 1.0169, 1.0456 and 1.057, 1.0151, 1.0138, respectively.

(8) $$PDI=\frac{M_{W}}{M_{N}}$$

For the bridge deck, in contrast to the SBS molecular weight before aging, the molecular weight of bitumen from BD-UL and BD-BL ranged from 10,000 to 14,000 and obviously decreased. This indicated that the upper and bottom layers were both aged, but the aging degree was different. The *M_n_* and *M_w_* of bitumen from the upper layer were lower than those of bitumen from the bottom layer. This meant that, in contrast to bitumen from the bottom layer, a large part of SBS molecules of bitumen from the upper layer were degraded into small molecules. The higher PDI for bitumen from the upper layer indicated a broader molecular weight distribution. This showed that the aging degree of bitumen from the upper layer was more serious than that of bitumen from the bottom layer under the effect of load and the environment.

For the traffic lane, results showed that the aging degree of bitumen from the three layers was slightly different. The minimal *M_n_* and *M_w_* values and the maximum PDI value suggested that the aging degree of bitumen from the upper layer was significantly more serious than that of bitumen from the other two layers. However, as can be seen in Figure 2, *M_n_* and *M_w_* of bitumen from the middle layer was slightly higher than that of bitumen from the bottom layer. The PDI of bitumen from the middle layer was slightly less than that of bitumen from the bottom layer, which indicated that the aging degree of the bottom layer was more serious. This could be due to cracks caused by the aging of the upper layer, where moisture and air can flow into the pavement structure layer. Oxygen and moisture accumulate in the bottom layer and react with bitumen, which can result in breaking of the bitumen molecular chain in the bottom layer. Therefore, the aging degree of bitumen from the bottom layer is more serious than that of bitumen from the middle layer.

For the ramp, the three indexes of the different layers were ranked as *M_n_*: the upper layer < the middle layer < the bottom layer; *M_w_*: the upper layer < the middle layer < the bottom layer; and PDI: the upper layer > the middle layer > the bottom layer. This meant the aging degree of bitumen from the upper layer was the most serious, followed by the middle layer, and finally the bottom layer.

#### 3.1.2. GPC Curves of Aged Bitumen

**Figure 3:**
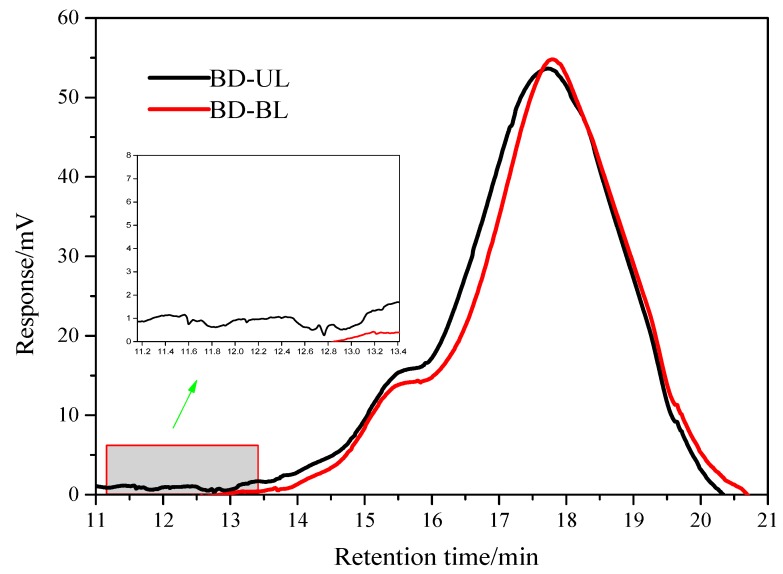
GPC curves of bitumen from BD-UL and BD-BL.

Figure 3 shows the GPC test results for the upper and bottom layers of the bridge deck. It can be seen that the trend of the two curves was basically the same, except that the size of the peak area was different at some specific positions. The bitumen used for the bridge deck was SBS-modified bitumen. As can be seen in Figure 3, regardless of the GPC curves of bitumen from BD-UL or that of bitumen from BD-BL, the SBS characteristic peak that should appear at about 12 min almost disappeared, indicating that the SBS modifier degradation of both was serious. At this time, judging the aging degree by characteristic peaks would not be reliable, so the LMS values of bitumen were used to judge the degree of aging. According to the calculation method (Figure 1), the LMS value of bitumen from BD-UL was calculated as 25.32, and the LMS value of bitumen from BD-BL was 20.82. This dramatic conclusion could be due to the fact that naphthenic and polar aromatics form more asphaltenes during aging, while the size of the asphaltene structure also increases [33].

**Figure 4:**
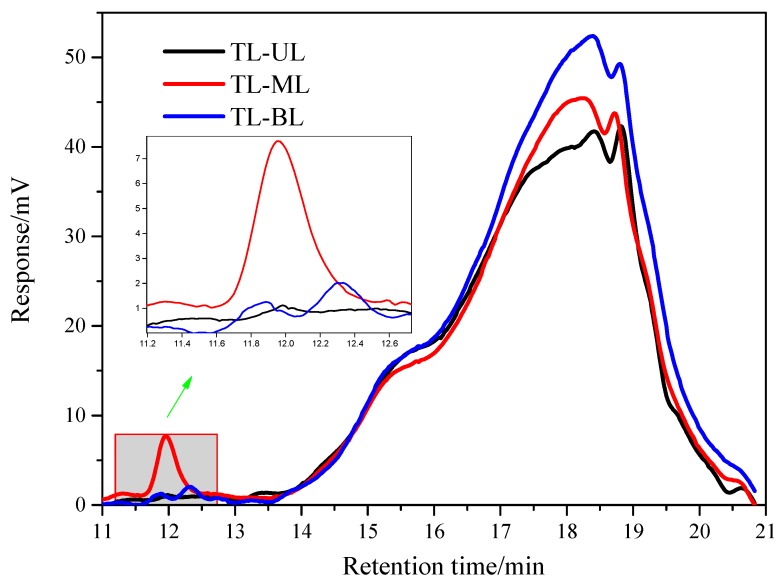
GPC curves of bitumen from TL-UL, TL-ML, and TL-BL.

Figure 4 shows the GPC test results of different layers of the traffic lane. It can be seen that the general trend of the three curves was consistent. At 12 min, the TL-ML layer showed a sharp peak and the peak of TL-BL was less intense than TL-ML, while the peak of TL-UL disappeared completely. This showed that TL-UL had the most serious aging, and the macromolecular material was broken completely. The peak in TL-ML indicated that the macromolecules were much less broken than TL-UL and TL-BL. At 18 min, the intensity of the TL-UL peak was significantly less than that of TL-ML and TL-BL, probably because the degradation of bitumen and SBS modifier from TL-UL was severe, and the macromolecule ruptured to form small molecules. However, the loss of small molecules in the upper layer was more serious under the influence of vehicle load and the environment. Therefore, the intensity of the peak here was weaker than that of bitumen from TL-ML and TL-BL. Overall, the aging degree of bitumen in the traffic lane was TL-UL > TL-BL > TL-ML.

**Figure 5:**
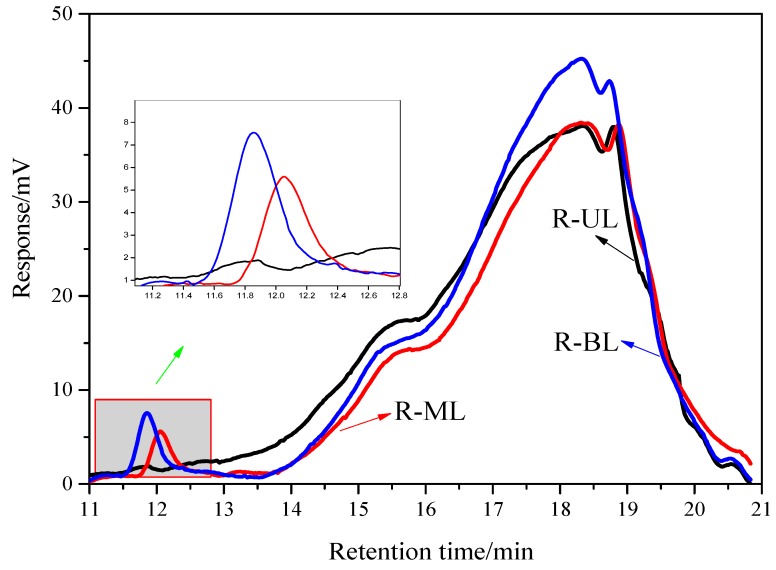
GPC curves of bitumen from R-UL, R-ML, and R-BL.

Figure 5 shows the GPC curve results for different layers of the ramp. In combination with the GPC curves, it can be seen that the general trend of the three curves was consistent. At 12 min, the peak that should have appeared here completely disappeared, indicating that the aging degree of bitumen from R-UL was the most serious. R-ML and R-BL showed sharp peaks here, and the peak of R-BL was sharper, indicating that the degradation degree of SBS modifier of the bitumen sample from R-BL was weaker, and the aging degree of bitumen was lower. As can be seen from Figure 5, the intensity of the R-BL peak was slightly higher than that of bitumen from R-UL and R-ML at 18 min, which could be due to the migration of a part of the small molecule material generated by the aging of the upper R-ML to the R-BL under the action of water flow. In summary, the aging degree of bitumen in the ramp was R-UL > R-ML > R-BL.

### 3.2. Composition Change Analyses of Bitumen

#### 3.2.1. Bitumen of Bridge Deck

**Figure 6:**
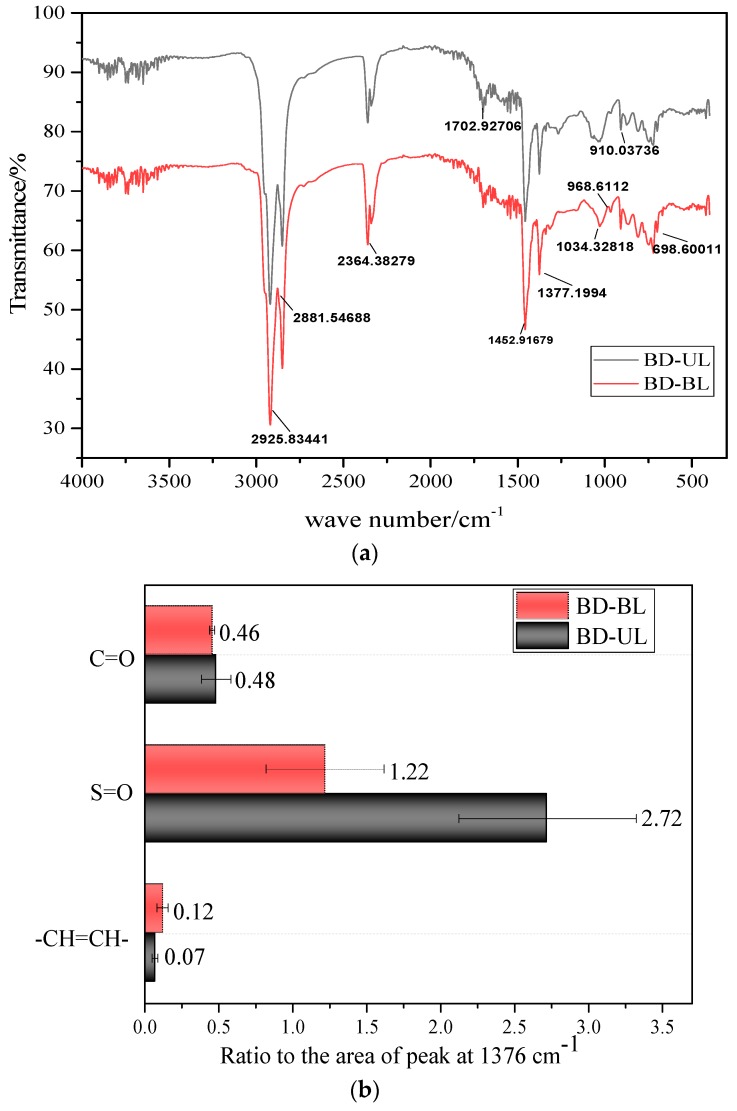
Composition changes of bitumen from BD-UL and BD-BL. (**a**) Infrared spectra; (**b**) quantitative analysis diagram.

Figure 6 shows the infrared spectrums and quantitative analysis diagrams of bitumen from different structural layers of the bridge deck. It can be seen from Figure 6 that the absorption peaks of samples from BD-UL and BD-BL appeared near 966 cm^−1^ and 698 cm^−1^, which were the two characteristic peaks of SBS modifier. The peak at 966 cm^−1^ and 698 cm^−1^ was the characteristic peak of C–C bond in butadiene and styrene, respectively. The absorption peak near 966 cm^−1^ of sample from BD-UL was weaker, which could be due to the direct contact of the SBS modifier in the upper layer with the environment, so the degradation of SBS was more serious. The absorption peak at 910 cm^−1^ was the absorption peak of benzene ring C–H vibration, reflecting that other components were substituted on the benzene ring of bitumen. The intensity of the absorption peak at 910 cm^−1^ of sample from BD-UL was slightly higher than that of the sample from BD-BL. The results showed that the substitutions of benzene ring in bitumen occurred more frequently in the sample from BD-UL. The absorption peaks of the sulfoxide group (S=O) of the samples from BD-UL and from BD-BL were near 1030 cm^−1^, indicating that both of them had aged, and the peak strength of sample from BD-UL was higher than that of the sample from BD-BL. The peak of 1600–1700 cm^−1^ was caused by the oxygen absorption of unsaturated carbon chain. The peak of the sample from BD-UL produced more peaks here, and the intensity of the peak was larger, indicating that there were more oxygen absorption reactions and more serious aging of sample from BD-UL. The comprehensive analyses showed that the aging degree of bitumen was BD-UL > BD-BL.

#### 3.2.2. Bitumen of Traffic Lane

**Figure 7:**
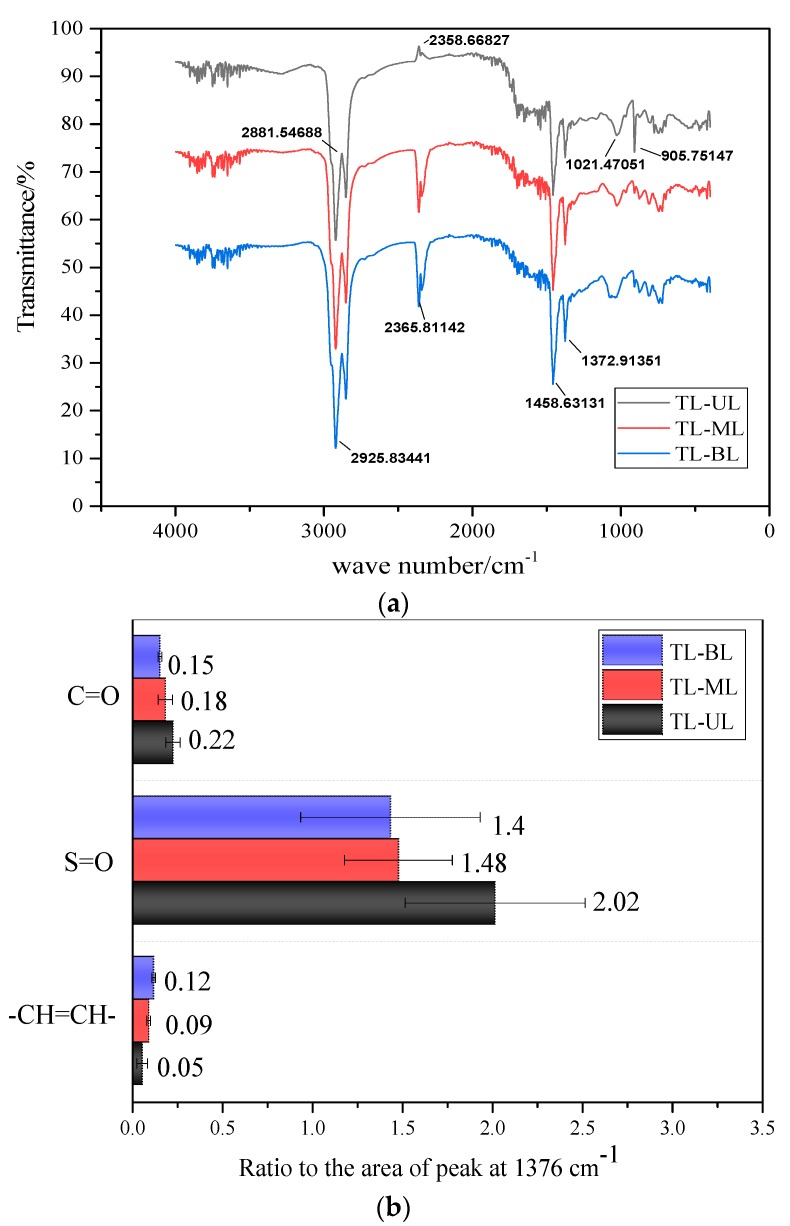
Composition changes of bitumen from TL-UL, TL-ML and TL-BL. (**a**) Infrared spectra; (**b**) quantitative analysis diagram.

According to the spectra shown in Figure 7, the intensity of the three absorption peaks at 1030 cm^−1^ and 1700 cm^−1^ of the upper layer was significantly higher than those of the middle layer and the bottom layer, indicating that the aging degree of bitumen from the upper layer was much greater than that of bitumen from the middle layer and the bottom layer. In contrast to the aging degree of bitumen from the middle layer and the bottom layer, the absorption peak intensity of the C–O bond formed by the oxygen absorption of unsaturated carbon chain at 1700 cm^−1^ of bitumen from the middle layer was slightly higher than that of bitumen from the bottom layer. The sulfoxide group absorption peak at 1030 cm^−1^ was also higher than that of bitumen from the bottom layer. Therefore, the aging degree of bitumen from the middle layer was slightly higher than that of bitumen from the bottom layer. According to the analyses of the infrared spectra, the aging degree of bitumen from the traffic lane was upper layer > middle layer > bottom layer.

#### 3.2.3. Bitumen of Ramp

**Figure 8:**
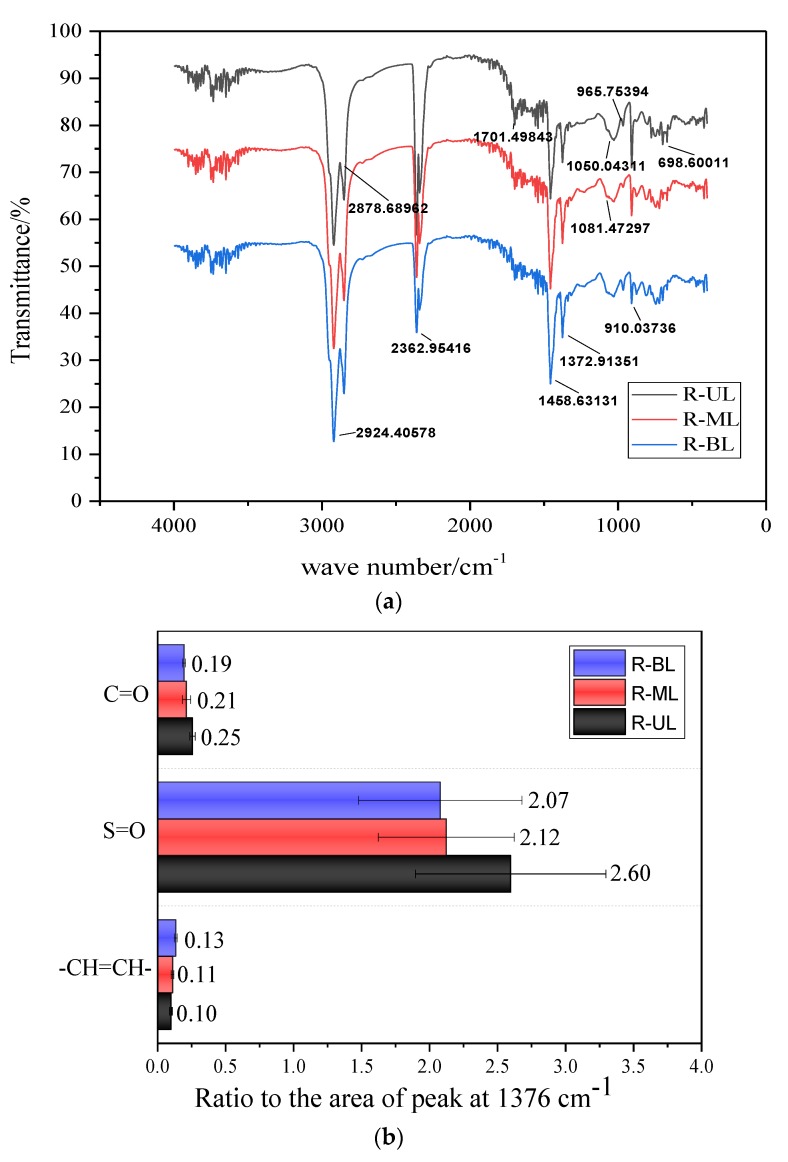
Composition changes of bitumen from R-UL, R-ML and R-BL. (**a**) Infrared spectra; (**b**) quantitative analysis diagram.

As shown in Figure 8, the infrared spectra of bitumen from the upper, middle, and bottom layers in the ramp were analyzed and compared. The absorption peaks of 966 cm^−1^ and 698 cm^−1^ indicated that SBS-modified bitumen was used in the ramp. The absorption peak at 1700 cm^−1^ indicated that bitumen began to absorb oxygen gradually, and the curves of samples from R-ML and R-BL decreased less before the absorption peak of 1700 cm^−1^. However, the curve of the sample from R-UL decreased sharply when it appeared at the peak. The sulfoxide group bond (S=O) (1030 cm^−1^) was present in the three samples. In contrast to the area of the peaks of the three samples, it was found that R-UL had the largest peak at 1030 cm^−1^, R-ML was the next, and the area of the peak of R-BL at 1030 cm^−1^ was the smallest. The results showed that there were more oxygen absorption aging reactions and more serious aging degree of bitumen from the upper layer. Therefore, the aging degree of bitumen from the ramp was R-UL > R-ML > R-BL, that is, the upper layer > the middle layer > the bottom layer.

According to the analyses of the aging degree of bitumen from the upper, middle, and bottom layers of the three road sections, the aging of bitumen from the upper layer was more serious than that of bitumen from the middle and lower layers because the upper layer was directly affected by the vehicle load and the environment. The oxidation of the unsaturated carbon chain and the sulfur element in bitumen was caused by the oxygen absorption reaction during the aging process, which resulted in the corresponding characteristic peaks.

### 3.3. Viscoelastic Behavior Analyses of Bitumen

#### 3.3.1. Bitumen of Bridge Deck

**Figure 9:**
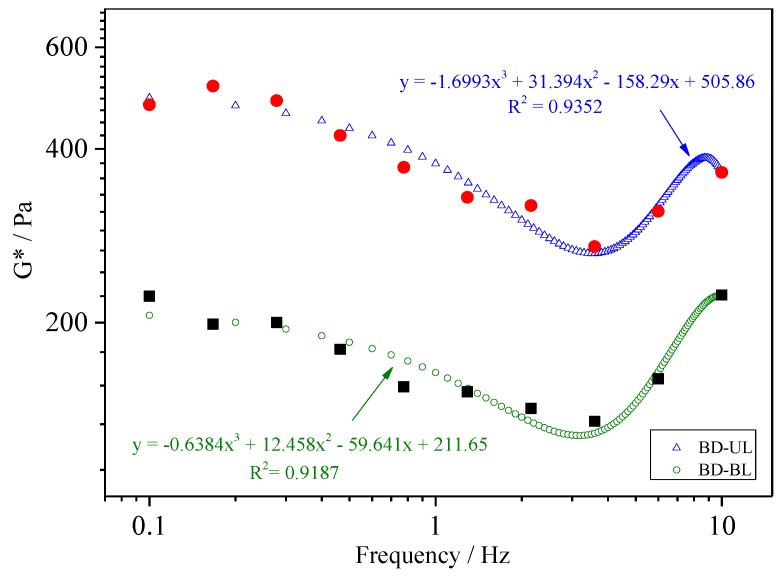
Complex shear modulus of bitumen from BD-UL and BD-BL.

Complex shear modulus can be used to evaluate the stiffness of bitumen. For the bridge deck, it can be seen from Figure 9 that the complex shear modulus of bitumen from BD-UL and BD-BL showed a slight downward trend in the low frequency state, while there was an increasing trend in the high frequency state. The sample conformed to the general trend of low to high growth of the complex shear modulus from low to high frequency. In both low and high frequency states, the complex shear modulus of bitumen from BD-UL was always higher than that of bitumen from BD-BL. This indicated that bitumen from BD-UL was harder than that from BD-BL, proving that the aging of bitumen from BD-UL was more serious than that of bitumen from BD-BL.

#### 3.3.2. Bitumen of Traffic Lane

**Figure 10:**
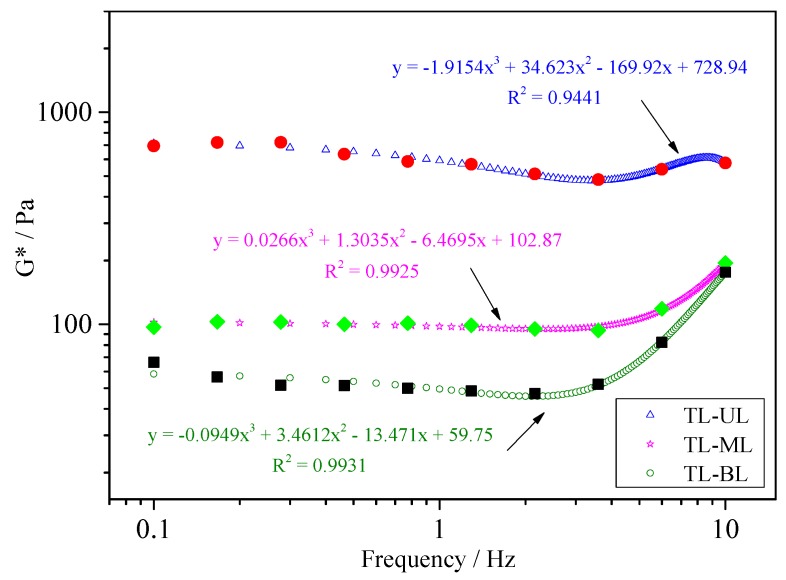
Complex shear modulus of bitumen from TL-UL, TL-ML, and TL-BL.

For the traffic lane, it can be seen from Figure 10 that complex shear modulus curves of bitumen from TL-UL, TL-ML, and TL-BL showed an upward trend with the increase in frequency. The order of the complex shear modulus in both low and high frequency states was TL-UL > TL-ML > TL-BL, indicating that bitumen from the traffic lane conformed to the aging degree, that is, the upper layer > the middle layer > the lower layer.

#### 3.3.3. Bitumen of Ramp

**Figure 11:**
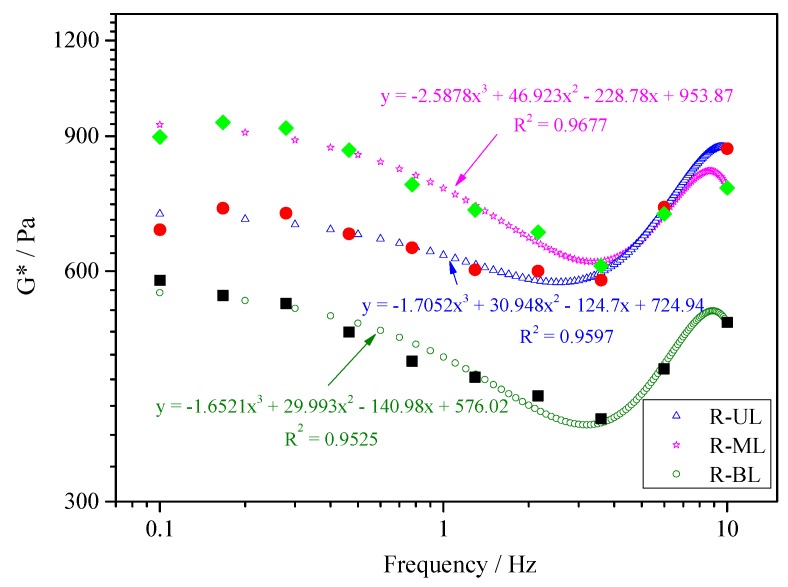
Complex shear modulus of bitumen from R-UL, R-M, and R-BL.

For the ramp, it can be seen from Figure 11 that the order of the complex shear modulus was R-ML > R-UL > R-BL in the low frequency ranges and R-UL > R-ML > R-BL in the high frequency ranges. The complex shear modulus of bitumen from the middle layer in the low frequency region was higher than that of bitumen from the upper layer, indicating that bitumen from the middle layer was harder than that from the upper layer in the high temperature environment. The complex shear modulus of bitumen from the middle layer in the high frequency ranges was lower than that of bitumen from the upper layer, indicating that bitumen from the middle layer was more flexible than that from the upper layer in the low temperature surroundings. This indicated that the performance of bitumen from the middle layer was better than that of bitumen from the upper layer in both low and high temperature conditions, that is, the aging degree of bitumen in the ramp was upper layer > middle layer > lower layer.

#### 3.3.4. Phase Angle of Different Bitumen

**Figure 12:**
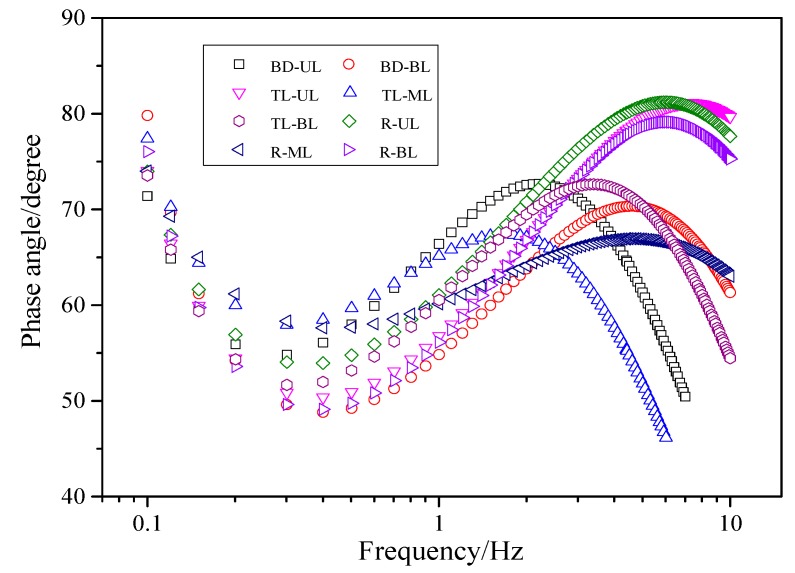
Phase angles of bitumen in different bituminous pavement structures.

It can be seen from Figure 12 that the phase angles of the samples were U-shaped. The phase angle is an indicator of the viscoelasticity of the materials. At medium–high temperature, the bitumen was in the region transitioning from a high elastic state to a viscous flow state. When the bitumen sample was approaching the viscous flow state, the phase angle could increase and approach 90 °C. At the same time, when the temperature was high and the load frequency was low, the phase angle decreased with the increase in temperature due to the influence of the mineral skeleton. When the load frequency was lower, the phase angle started to rise with the decrease in frequency due to the more complicated viscoelastic behavior of the bitumen in the phase transition stage. At lower temperatures and higher load frequencies, the phase angle decreased rapidly, and bitumen changed from a viscous state to a high-elastic state.

### 3.4. Microscopic Morphology Analyses of Bitumen

#### 3.4.1. Bitumen of Bridge Deck

**Figure 13:**
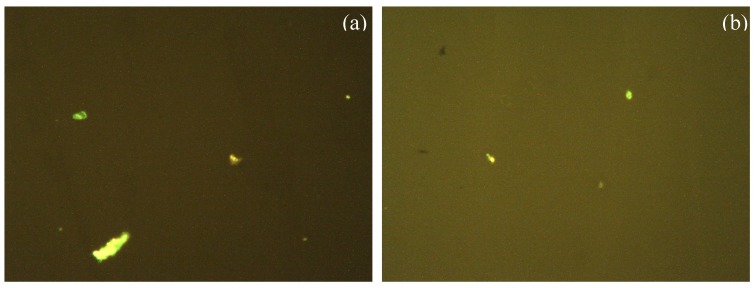
Fluorescence microscope (FM) pictures of bitumen from BD-UL and BD-BL (100×): (**a**) BD-UL and (**b**) BD-BL.

Figure 13 shows the fluorescence pictures of bitumen extracted from the bridge deck. The original bitumen was black in the fluorescence microscopy image because it was not excited by fluorescence, while the SBS modifier was bright yellow. It can be seen from Figure 13 that the SBS modifiers from BD-UL and BD-BL were in the dispersed phase of the particles, indicating that bitumen had aged due to the influence of load and the environment. However, the difference between the two pictures was very small, meaning the aging degree of bitumen from the upper and middle layers in the bridge deck could not be evidently distinguished from the fluorescence pictures.

#### 3.4.2. Bitumen of Traffic Lane

**Figure 14:**
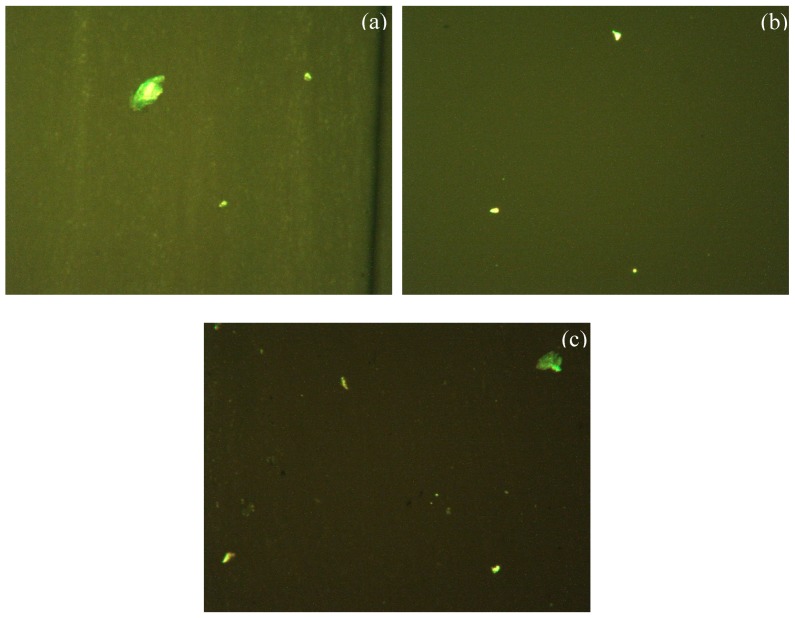
FM pictures of bitumen from TL-UL, TL-ML, and TL-BL (100×): (**a**) TL-UL; (**b**) TL-ML; and (**c**) TL-BL.

It can be seen from Figure 14 that the SBS modifier particles of bitumen from TL-UL and TL-ML were sparse, which indicated that the aging degree of bitumen from the upper and lower layers of the lane was relatively serious. However, the aging degree of the two bitumen could not be evidently distinguished in the fluorescence pictures due to the very close number of particles and dispersion state. The bitumen from TL-BL showed a continuous phase, and the picture was doped with SBS modifier, proving that bitumen from TL-BL possessed the lowest aging degree.

#### 3.4.3. Bitumen of Ramp

**Figure 15:**
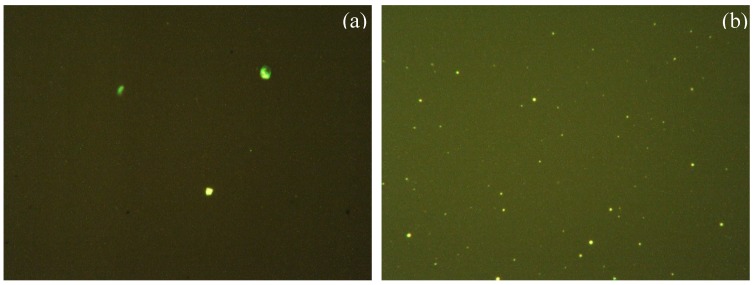

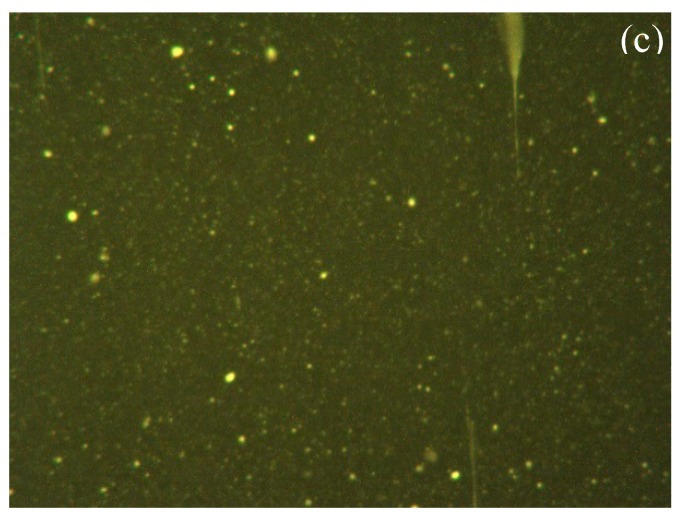
FM pictures of bitumen from R-UL, R-ML, and R-BL (100×): (**a**) R-UL; (**b**) R-ML; and (**c**) R-BL.

The SBS modifier can absorb the light components in the original bitumen to form a continuous phase shortly after the SBS modifier is added to bitumen. It can be seen from Figure 15 that, after long-term aging, the continuous phase formed by the SBS modifier had varying degrees of damage. In bitumen from R-UL, the SBS modifier was in the form of granular-dispersed phase, and SBS-modified bitumen became a single-phase continuous structure. The quantity of SBS modifier particles in bitumen from R-ML increased and existed in the state of network structure compared with bitumen from R-UL. The number of bitumen from R-BL reached the maximum, indicating that the SBS modifier from bitumen from R-BL had the lowest degradation degree. Therefore, the aging degree of bitumen in the ramp was R-UL > R-ML > R-BL, that is, the upper layer > the middle layer > the bottom layer.

### 3.5. Comparison of Results with Different Bituminous Pavement Structure

The aging rules characterized by different analytical methods showed that, while the aging degree of SBS-modified bitumen from different structural layers in service was different, they basically met the same order of aging, that is, upper layer > middle layer > bottom layer. The results showed that using different analytical methods to analyze the aging rules of the same sample could give slightly different conclusions. The conclusions of the aging changes obtained by different characterizations are detailed in Table 2.

## 4. Conclusions and Recommendations

In this work, the aging characteristics of bitumen from different structural layers in bituminous pavement were characterized and analyzed, and differences in the aging degree of bitumen in different structural layers were analyzed from their chemical compositions and microstructures. The following conclusions were drawn:

(1) Bitumen from the upper and middle layers of the bridge deck was aged. The aging changes conformed to the rule that the bitumen macromolecular chain breaks into small molecules, the small molecule content increases, and the molecular weight width therefore increases. SBS modifiers existed in the particle dispersed phase after aging. The aging type was oxygen absorption aging, which was mainly at peaks of 1600–1700 cm^−1^. According to the analysis results of GPC and FTIR, the aging degree of bitumen from the upper layer of the bridge deck was more serious than that of bitumen from the middle layer.

(2) The aging degree of bitumen from the three surface layers of the traffic lane was slightly different. According to the results of GPC, bitumen from the upper layer had the smallest molecular weight and the most severe aging, bitumen from the bottom layer had less aging, and bitumen from the middle layer had the lowest degree of aging. However, according to FTIR, DSR, and FM, bitumen from the bottom layer of the lane had the lowest degree of aging as indicated by oxygen aging, the lowest complex shear modulus at different frequencies, and the continuous phase structure. This could be due to the migration of small molecular chains formed by the aging of bitumen from the upper layer to the middle layer, which had not yet migrated to the bottom layer.

(3) For bitumen from the upper, middle, and bottom layers of the ramp, different testing methods could obtain the same changes in aging degree in the different surface layers of the ramp, and they were upper layer > middle layer > bottom layer. This was because the traffic volume at the ramp would be small, and the aging would gradually expand from the upper layer to the bottom layer of the bituminous pavement structure in service.

(4) The aging changes characterized by different analytical methods showed that the aging degree of bitumen in different layers of the bituminous pavement structure in service was different, but they met the same order of aging degree, that is, upper layer > middle layer > bottom layer. After aging, the microstructures and the compositions of bitumen changed, which could affect properties of pavement structures. When diseases such as longitudinal joints occur, maintenance measures should be taken in time to avoid further deepening of aging. Due to the inevitable early aging of the upper layer, further research and optimization is recommended on the structure and material aspects of the upper layer of bituminous pavements.

## Figures and Tables

**Table 1:** Descriptions of different bituminous pavement structure and core samples.

Nos.	Samples		Pavement Thickness	Service Years	Pavement Diseases	Core Sample Description	Pictures of Pavements and Core Samples
	Bituminous Pavement Structures	Sample Labels					
1	Bridge deck	Bridge deck, upper layer of bituminous pavement (BD-UL)	5	15	Longitudinal cracks	The core sample is complete; there are tiny cracks on the surface, and there is no crack inside.	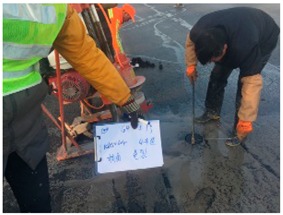 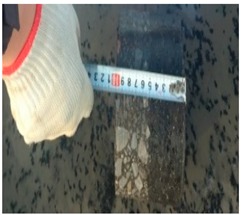
2		Bridge deck, bottom layer of bituminous pavement (BD-BL)	5				
3	Traffic lane	Traffic lane, upper layer of bituminous pavement (TL-UL)	4	15	Longitudinal cracks	The core sample is intact, and the interlayer adhesion is good.	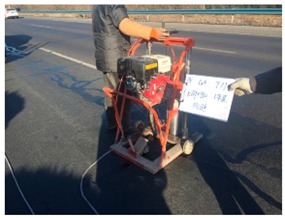 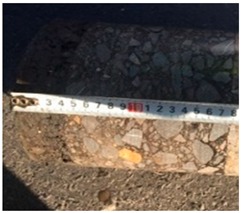
4		Traffic lane, middle layer of bituminous pavement (TL-ML)	7				
5		Traffic lane, bottom layer of bituminous pavement (TL-BL)	7				
6	Ramp	Ramp, upper layer of bituminous pavement (R-UL)	3	15	Mesh cracks	The core sample is intact; there are surface cracks, and the adhesion between the two layers is poor.	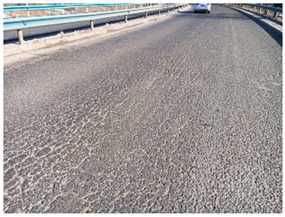 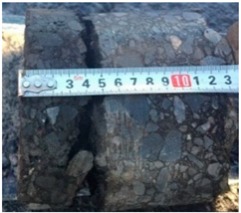
7		Ramp, middle layer of bituminous pavement (R-ML)	5				
8		Ramp, bottom layer of bituminous pavement (R-BL)	5				

**Table 2:** Aging degree sequences of bitumen with different analytical methods.

Bituminous Pavement	Analytical Methods			
	GPC	FTIR	DSR	FM
Bridge deck	upper > middle	upper > middle	middle > upper	middle ≈ upper
Traffic lane	upper > bottom > middle	upper > middle > bottom	upper > middle > bottom	upper ≈ middle > bottom
Ramp	upper > middle > bottom	upper > middle > bottom	upper > middle > bottom	upper > middle > bottom
